# KYNA/Ahr Signaling Suppresses Neural Stem Cell Plasticity and Neurogenesis in Adult Zebrafish Model of Alzheimer’s Disease

**DOI:** 10.3390/cells10102748

**Published:** 2021-10-14

**Authors:** Tohid Siddiqui, Prabesh Bhattarai, Stanislava Popova, Mehmet Ilyas Cosacak, Sanjeev Sariya, Yixin Zhang, Richard Mayeux, Giuseppe Tosto, Caghan Kizil

**Affiliations:** 1 German Center for Neurodegenerative Diseases (DZNE) within Helmholtz Association, Tatzberg 41, 01307 Dresden, Germany; tohid.siddiqui@dzne.de (T.S.); prabesh.bhattarai@dzne.de (P.B.); stanislava.popova@dzne.de (S.P.); mehmet.cosacak@dzne.de (M.I.C.); 2 The Department of Neurology, The Taub Institute for Research on Alzheimer’s Disease and the Aging Brain, Columbia University Irving Medical Center, 630 West 168th Street, New York, NY 10032, USA; ss5505@cumc.columbia.edu (S.S.); rpm2@cumc.columbia.edu (R.M.); gt2260@cumc.columbia.edu (G.T.); 3 B-CUBE, Center for Molecular Bioengineering, TU Dresden, Tatzberg 41, 01307 Dresden, Germany; yixin.zhang1@tu-dresden.de

**Keywords:** zebrafish, Alzheimer’s disease, neurogenesis, regeneration, neural stem cell, kynurenic acid, plasticity, transcriptome-wide association study, proliferation

## Abstract

Neurogenesis decreases in Alzheimer’s disease (AD) patients, suggesting that restoring the normal neurogenic response could be a disease modifying intervention. To study the mechanisms of pathology-induced neuro-regeneration in vertebrate brains, zebrafish is an excellent model due to its extensive neural regeneration capacity. Here, we report that Kynurenic acid (KYNA), a metabolite of the amino acid tryptophan, negatively regulates neural stem cell (NSC) plasticity in adult zebrafish brain through its receptor, aryl hydrocarbon receptor 2 (Ahr2). The production of KYNA is suppressed after amyloid-toxicity through reduction of the levels of Kynurenine amino transferase 2 (KAT2), the key enzyme producing KYNA. NSC proliferation is enhanced by an antagonist for Ahr2 and is reduced with Ahr2 agonists or KYNA. A subset of Ahr2-expressing zebrafish NSCs do not express other regulatory receptors such as *il4r* or *ngfra*, indicating that *ahr2*-positive NSCs constitute a new subset of neural progenitors that are responsive to amyloid-toxicity. By performing transcriptome-wide association studies (TWAS) in three late onset Alzheimer disease (LOAD) brain autopsy cohorts, we also found that several genes that are components of KYNA metabolism or AHR signaling are differentially expressed in LOAD, suggesting a strong link between KYNA/Ahr2 signaling axis to neurogenesis in LOAD.

## 1. Introduction

Alzheimer’s disease (AD) is the most widespread neurodegenerative disease [1,2,3,4,5,6]. Manifestations of AD are heterogenous in onset and pathology. This variability suggests that multiple factors and cell types are involved in the cause and progression of AD. Historically, AD has been investigated from a neurocentric perspective focused on restoring synaptic connectivity; this approach was challenged by the identification of multiple cell types that partake in disease etiology [7,8,9]. The causal relationship between immune cells, astrocytes, oligodendrocytes, and endothelial cells [1,10,11,12] necessitates investigations on less studied aspects of AD. For instance, inflammatory processes are closely related to AD from a cellular and genetic perspective, as chronic inflammation has detrimental effects on neuronal health, and several genetic variants of genes that are associated with AD have predominant functions in immune cells, such as *APOE* or *TREM2* [2,13,14,15,16]. Another important player that might have profound effects on AD is neurogenesis from neural stem cells (NSCs) [17,18,19]. Although the immune and neuronal aspects in AD receive more attention, there is still a need to address NSCs and their contribution to the disease [19,20,21,22]. Defects in neurogenesis might be a cause or modifier of AD, and neurogenesis on demand—neural regeneration—could be used to counteract the disease by enhancing the resilience of the brain to cope better with neurodegeneration [19,23,24,25,26,27]. 

To investigate the “regenerative ability” in AD conditions, we generated an experimental model of amyloidosis in adult zebrafish brain [26,28,29]. To determine the changes in gene expression in neural stem cells and neurons, we performed transcriptome profiling analysis by comparing control and Aβ42-injected brains. Our analyses showed that Interleukin-4 (IL4) is among the most upregulated genes in fish brain after Aβ42-injection. Based on available tools, we observed that compared to controls, Aβ42-injected brains express more IL4 and also, IL4 was sufficient to induce NSC proliferation and regenerative neurogenesis [26]. One of the pathways regulated by IL4 in adult zebrafish brain was tryptophan metabolism, which functionally regulated neural stem cell plasticity and neurogenesis through serotonin signaling [30]. In our analyses, we also found that the kynurenine branch of the tryptophan metabolism was also regulated by IL4 to reduce the expression of key enzymes that convert tryptophan to kynurenic acid (KYNA). In this manuscript, we investigate the role of KYNA and its receptor in regulating NSC plasticity. Here, we demonstrate that amyloid toxicity reduces the levels of kynurenine aminotransferase II (AADAT/KAT2), and KYNA is a negative regulator of neural stem cell proliferation and neurogenesis by acting through its receptor Aryl hydrocarbon receptor 2 (Ahr2 in zebrafish, AHR in humans). Adult zebrafish neural stem cells respond to amyloid toxicity through different subtypes of NSCs, namely through direct regulation by Interleukin-4 (IL-4) and by brain-derived neurotrophic factor (BDNF) via its receptor nerve growth factor receptor (NGFRA). Here, we present a new subtype of NSC that is responsive to KYNA.

## 2. Materials and Methods

### 2.1. Ethics Statement

All animal experiments were performed in accordance with the animal experimentation permit from Referat 25/26 (Veterinärwesen, Pharmazie, und GMP), Landesdirektion Sachsen Germany and TU Dresden (Kommission für Tierversuche). Zebrafish maintenance was according to the published guidelines [31] and EU Directive 2010/63 Article 33 and Annex III (permit numbers: TVV-31/2019 and TVV-52/2015).

### 2.2. Animal Maintenance

All the experiments were conducted on adult zebrafish from wildtype AB strain and Tg (*her4.1*:GFP); aged between 6–8 months and both sexes were used. Fish from the same clutches were randomly assigned in different experimental groups.

### 2.3. Tissue Preparation

Animals were euthanized at indicated time points. Dissected heads were fixed in 4% paraformaldehyde (Merck, Darmstadt, Germany). After fixation, they were washed three times with 0.1 M phosphate buffer (pH 7.4) and transferred into 20% Sucrose (Merck, Darmstadt, Germany)/20% ethylenediaminetetraacetic acid (EDTA, Merck, Darmstadt, Germany) in 0.1 M phosphate buffer (pH 7.4) overnight (at 4 °C) for decalcification and cryoprotection. Tissue was embedded in cryoprotectant sectioning resin that consisted of 20% Sucrose (Merck, Darmstadt, Germany)/7.5% Gelatin (Merck, Darmstadt, Germany). Tissues were stored at −80 °C. Cryosectioning was performed to generate thin sections (14 μm). Tissue sections were stored at −20 °C.

### 2.4. Cerebroventricular Microinjection

Cerebroventricular microinjection (CVMI) in adult zebrafish brain were performed as previously described [26,32,33]. Briefly, animals were anesthetized until the opercular movement ceased, followed by a minor incision in the skull above the optic tectum using a 30 G needle, without injuring the brain tissue. Using a fine glass capillary (WPI, Sarasota, FL, USA), the injection solution was applied through the incision in the direction of the telencephalon. PBS (1 μL), Aβ42 (1 μL, 20 μM), IL4 (1 μL, 10 μg/mL, ThermoFisher Scientific, Darmstadt, Germany), BNF (1 μL, 10μM, Sigma Aldrich, St. Louis, MO, USA), Ahr antagonist (1 μL, 6 μM, Merck, Darmstadt, Germany), and KYNA (1 μL, 10 μM, Sigma Aldrich, St. Louis, MO, USA).

### 2.5. In Situ Hybridization and Immunohistochemistry

For IHC, the sections were thawed for 20 min and post fixed with methanol for 15 min, followed by washing steps in PBS with 0.03% Tween-20 (Merck, Darmstadt, Germany) (PBSTx). Desired primary antibodies were applied on the slides and incubated overnight at 4 °C. After washing thrice with PBSTx, slides were incubated at room temperature with the secondary antibodies for 3 h. DAPI (200 μM, Invitrogen, Waltham, MA, USA) was included during incubation with the secondary antibody. Finally, slides were mounted using aquamount (Thermo Fisher Scientific, Darmstadt, Germany). Tissue was stored at 4 °C. For in situ hybridization, digoxigenin (DIG, Roche, Basel, Switzerland)-labeled cyp1b1 probe was generated with DIG RNA labeling kit (Roche, Basel, Switzerland) as described [26,34]. See Table S2 for further information.

### 2.6. Quantitative Real-Time PCR

Tg (*her4.1*:GFP) fish were used for the real time qPCR. Fish were anesthetized and the telencephalon was carefully dissected using ice-cold PBS. Using the neural tissue dissociation kit (Miltenyi Biotec, Bergisch Gladbach, Germany), cell were dissociated [26] and passed through a 40-μm cell strainer. To sort the live GFP positive cells, viability indicator (propidium iodide, Merck, Darmstadt, Germany) and GFP were used as markers. Total RNA was extracted from the sorted cell and reverse transcribed using the Superscript IV VILO Mastermix kit (Invitrogen, Carlsbad, CA, USA) according to the manufacturer’s instructions. CFX96 Real-Time System (BIORAD, Hercules, CA, USA) was used to perform the real time qPCR, with a 20 μL reaction volume comprising of SYBR Green I Master mix (Roche, Basel, Switzerland), 1 μM primers, and cDNA. Relative gene expression method was used to analyze the results, using beta-actin as the housekeeping gene. All primers used for qPCR are provided in Table S2.

### 2.7. Single Cell Sequencing and Analyses

RNA isolation from zebrafish telencephali, library preparation and bioinformatics analyses were performed as described [30,35]. GEO accession numbers: GSE74326 [26], GSE118577 [35] and GSE124162 [30].

### 2.8. Human Bulk RNA-Sequencing

We included in this study three human brain cohorts: (1) The Religious Orders Study and Memory and Aging Project (ROS/MAP); (2) the Mayo Clinic RNAseq cohort and (3) the Columbia University Hispanic Brain Bank cohort. Demographic, RNA-sequencing methods, expression quantification, and initial quality control are extensively described elsewhere [36]. (1) The sample characteristics of the ROS/MAP cohort subset studied here have been published in detail elsewhere [37]. Briefly, RNA sequencing data was analyzed from postmortem dorsolateral prefrontal cortexes (DLPFCs) of 595 subjects [37,38]. After quality control (QC; [36]), 583 subjects remained (AD = 354, non-AD = 229). (2) The Mayo RNAseq cohort has been described in detail elsewhere [39]; 148 subjects were included (AD = 80, non-AD = 68). Protocols were approved by the Mayo Clinic Institutional Review Board and all subjects or next of kin provided informed consent. (3) The Columbia University Hispanic Brain Bank cohort included temporal cortex (TCX) tissue from 45 unrelated self-reported Caribbean-Hispanic (CH) individuals. Cases were selected if age >50 years old, and neuropathologically defined Late-Onset Alzheimer’s Disease (LOAD) or control without any neuropathological diagnosis. The protocol was approved by the institutional review boards of the New York State Psychiatric Institute and Columbia University.

### 2.9. AD Transcriptome-Wide Association Studies (TWAS) and Meta-Analysis

Gene counts for all three cohorts were post-processed identically. Counts were read into R (v3.6.3, R Foundation, Indianapolis, IN, USA) for processing with edgeR [40] and limma/voom [41,42]. TMM normalization values (using edgeR calcNormFactors) and mean-variance derived observational-level weights were then calculated for linear modelling using limma/voom. For TWAS analyses, the limma package ‘lmFit’ function was used to model log2 (expected counts) as a linear function of pathological LOAD status, sequencing batch (brain bank source and flow cell included for Mayo), age, sex, RIN, postmortem interval (not available for the CH sample), percent of usable bases (not included for CH, as it was highly co-linear with RIN, and additional parameters were detrimental to model performance given small sample size), percent duplicated reads, median 3′ bias, and percent of mapped ribosomal bases. In CH models that included PSEN1 mutation carriers, a parameter was added to model its effect. Significance of the contrast between LOAD and non-LOAD status (LOAD vs. control for Mayo) was performed using empirical Bayes moderation (eBayes function). We meta-analyzed differential expression results from the three TWAS employing a fixed-effect model and using logFC and its standard error (SE) as inputs, consistently with previous studies of transcriptomics in LOAD [36,43,44]. The SE was calculated from the logFC values for each gene, separately in each cohort, and used for standard meta-analysis using the R package “rmeta”. LogFC and SE ensured that the direction of effect was taken into consideration when performing the meta-analysis. For dataset information see Table S1.

### 2.10. Imaging and Quantifications

ZEN software (blue edition, v1.0, Carl Zeiss, Jena, Germany) on a Zeiss fluorescent microscope with ApoTome or Zeiss AxioImager Z1 (Oberkochen, Germany) was used to acquire the fluorescence images, which were analyzed using ZEN (Carl Zeiss, Jena, Germany) or ImageJ (v1.8.0, NIH, Bethesda, MD, USA, https://imagej.nih.gov/ij/, accessed on 22 September 2021) software. Stereological quantifications were manually performed, examining one-fourth of the telencephalon sections, considering the region from caudal olfactory bulb until the rostral optic tectum. At least 3 animals with a minimum of 7 to 12 histological sections per animal were used for stereological analyses in all ISH and IHC stains unless otherwise stated.

### 2.11. Statistical Analyses 

The statistical evaluation was performed using Microsoft Excel (Microsoft Corporation, Redmond, WA, USA) and GraphPad Prism (version 8.02, GraphPad Sotware, San Diego, CA, USA). After estimating the statistical normality (using the Kolmogorov–Smirnov test calculator, https://www.socscistatistics.com/tests/kolmogorov/default.aspx, accessed on 22 September 2021) and variances (via Bartlett’s test) for experimental data sets, one-way ANOVA was used to analyze multiple data sets under the Bonferroni hypothesis and the Student’s *t*-test was performed for paired samples. Error bars shown indicate the SD, and asterisks indicate significance: * *p* < 0.05, ** *p* < 0.01, and *** *p* < 0.001. *p* > 0.05 is considered not significant (not indicated on the plots).

## 3. Results

**Figure 1:**
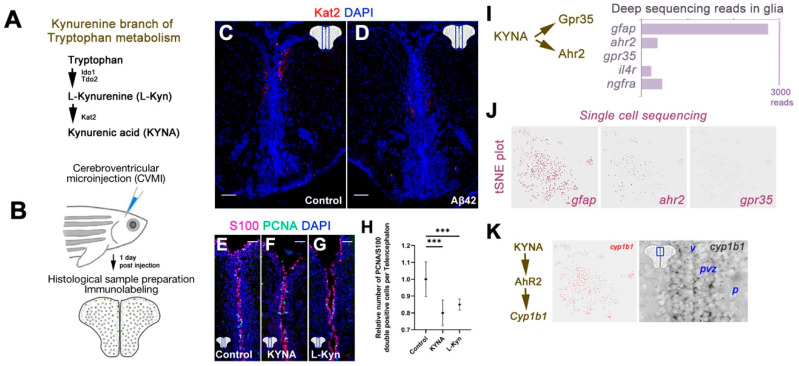
Kynurenic acid signaling is through Ahr2 in adult zebrafish brain. (**A**) Catabolic cascade from tryptophan to kynurenic acid. (**B**) Cerebroventricular microinjection paradigm. (**C**,**D**) Immunohistochemical staining for Kat2 in control (**C**) and Aβ42-injected (**D**) adult zebrafish brain sections. (**E**–**G**) Immunohistochemical staining for S100 and PCNA in control (**E**), KYNA-injected (**F**) and L-Kynurenine-injected (**G**) zebrafish brain sections. (**H**) Quantification of proliferating glial cells of conditions in (**E**–**G**). (**I**) Deep sequencing reads of KYNA receptors *ahr2* and *gpr35* as well as other receptors of neural stem cells, *il4r* and *ngfra*. Reads for *gfap* is for comparison. (**J**) tSNE plots from single cell sequencing in adult zebrafish telencephalon in progenitor cells. Expression of *gfap, ahr2* and *gpr35* shown. *ahr2* is the only receptor for KYNA in progenitor cells. (**K**) Ahr2 signaling regulates *cyp1b1* expression. *Cyp1b1* is present in progenitor cells as determined in single cell sequencing, and in situ hybridization. Scale bars equal 50 μm. Statistical calculations and values are provided in Table S1.

Tryptophan is converted to kynurenic acid (KYNA) through the activities of three enzymes: IDO1, TDO2 and KAT2 (Figure 1A). KAT2 catalyzes the final step that converts L-Kynurenine to KYNA. By using the cerebroventricular microinjection, we injected different metabolites and amyloid-beta42 (Aβ42) into adult zebrafish brains in this study (Figure 1B). We found that the levels of KAT2, which is expressed in the periventricular region of zebrafish brain in controls (Figure 1C), reduced after injection of Aβ42 (Figure 1D). Since Aβ42 enhances neural stem cell proliferation [26,30], we hypothesized that tryptophan metabolites could negatively affect proliferative outcome. By injecting KYNA and L-kynurenine and performing immunohistochemical staining for PCNA (proliferation marker) and S100β (glial cell marker) (Figure 1E–G), we found that proliferation reduced significantly (Figure 1H), suggesting that KYNA is a negative regulator of neural stem cell (NSC) proliferation in adult zebrafish brain. The two main receptors that KYNA binds to are GPR35 and AHR2 [45,46,47]. Our previous bulk and single cell sequencing results [26,30,35] showed that glial cells express *ahr2* to comparable levels to *il4r* and *ngfra*—two receptors we functionally showed to be relevant to proliferation, while gpr35 was not detectable (Figure 1I,J). To confirm this, we determined the expression of *cyp1b1*, a gene downstream to Ahr2 signaling, and found that *cyp1b1* is expressed in the ventricular and periventricular regions of the adult zebrafish telencephalon (Figure 1K).

**Figure 2:**
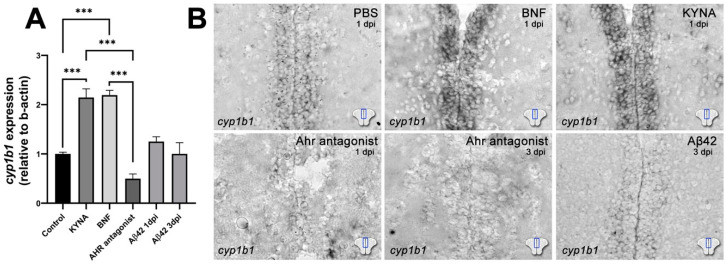
Ahr2-dependent *cyp1b1* expression is regulated by Aβ42 and KYNA in the adult zebrafish brain. (**A**) Normalized expression levels of *cyp1b1* in zebrafish telencephalon injected with control vehicle (PBS), KYNA, BNF, Ahr antagonist or Aβ42. (**B**) In situ hybridization for *cyp1b1* in control, BNF, KYNA, antagonist and Aβ42 injected brains. Note the reduction of expression with the Ahr antagonist and Aβ42 and increase with BNF or KYNA. Scale bars equal 50 μm. Statistical calculations and values are provided in Table S1.

**Figure 3:**
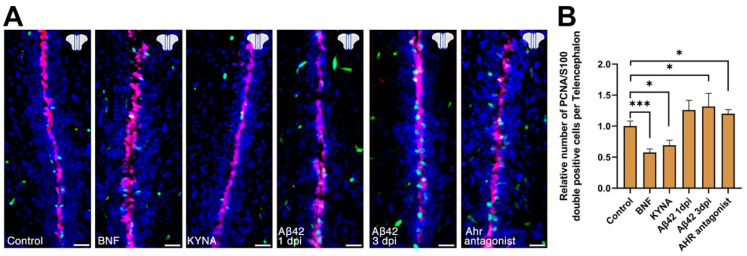
KYNA/Ahr2 signaling regulates neural stem cell proliferation in adult zebrafish brain. (**A**) Immunohistochemical stains for S100β (red) and PCNA (green) in control, BNF, KYNA, Aβ42 and Ahr antagonist injected brains. (**B**) Quantification of relative numbers of proliferating glial cells in conditions in A. Scale bars equal 50 μm. Statistical calculations and values are provided in Table S1.

To determine whether Ahr2 signaling can be modulated by KYNA, agonists and antagonists, we performed CVMI and determined the *cyp1b1* expression in the ventricular glial cells by quantitative RT-PCR and in situ hybridization (Figure 2). KYNA and Ahr2 agonist β-naphthoflavone (BNF) increased *cyp1b1* expression significantly, while the antagonist or Aβ42 did not show statistical significance in multiple comparison despite the individual replicate values show a trend of reduced *cyp1b1* expression (Figure 2A). To obtain spatially defined *cyp1b1* expression analyses, we performed in situ hybridization, which revealed that *cyp1b1* is expressed in the ventricular and periventricular regions of the adult zebrafish telencephalon, BNF and KYNA increased the expression levels significantly, Ahr antagonist reduced the expression, and Aβ42 reduced the expression in periventricular regions significantly (Figure 2B). We performed in situ analyses at 1 day post injection (dpi) for PBS, BNF, KYNA and Ahr antagonist (Figure 2B), and to show the continual reduced expression of *cyp1b1*, we also examined 3 dpi for Ahr antagonist and Aβ42 (Figure 2B). To determine whether modulation of Ahr2 signaling would affect neural stem cell (NSC) proliferation in adult zebrafish brains, we injected BNF, KYNA, Aβ42 and Ahr antagonist, performed immunostaining for S100β and PCNA, and quantified the proliferation response at 1 dpi and 3 dpi (Figure 3A). Compared to control brains, BNF and KYNA significantly reduced NSC proliferation, while Aβ42 and Ahr antagonist increased proliferation of NSCs (Figure 3B), indicating that KYNA negatively affects NSC plasticity through Ahr2 signaling.

**Figure 4:**
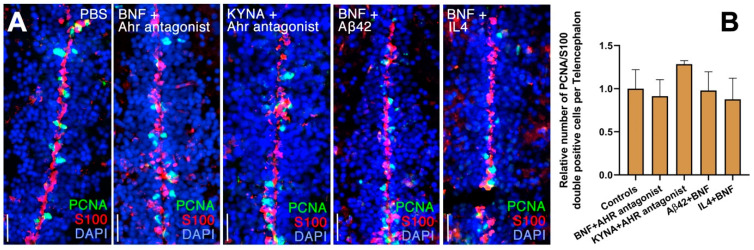
Ahr2-dependent suppression of neural stem cell plasticity is antagonized by neuroregenerative factors in adult zebrafish brain. (**A**) Immunohistochemical stains for S100β (red) and PCNA (green) in control (PBS), BNF+Ahr antagonist, KYNA+Ahr antagonist, BNF+Aβ42 and BNF+IL4 injected brains. (**B**) Quantification of relative numbers of proliferating glial cells in conditions in A. Scale bars equal 50 μm. Statistical calculations and values are provided in Table S1.

Next, we investigated whether the effects on NSC proliferation after activation of Ahr2 signaling through KYNA or BNF could be reversed by Ahr antagonist, Aβ42 or a key effector of amyloid toxicity in the zebrafish brain, Interleukin-4 (IL4), by performing immunohistochemical stains of proliferating NSCs (Figure 4A). When an activator (KYNA or BNF) and a suppressor (Aβ42, IL4 or antagonist) were injected at the same time, NSC proliferation did not change in comparison to control brains injected with PBS (Figure 4A), indicating that amyloid toxicity is suppressing Ahr2 signaling in adult zebrafish brain to allow for increased NSC proliferation. Additionally, cell proliferation also did not alter when the activator and the suppressor were co-injected (Figure 4B).

**Figure 5:**
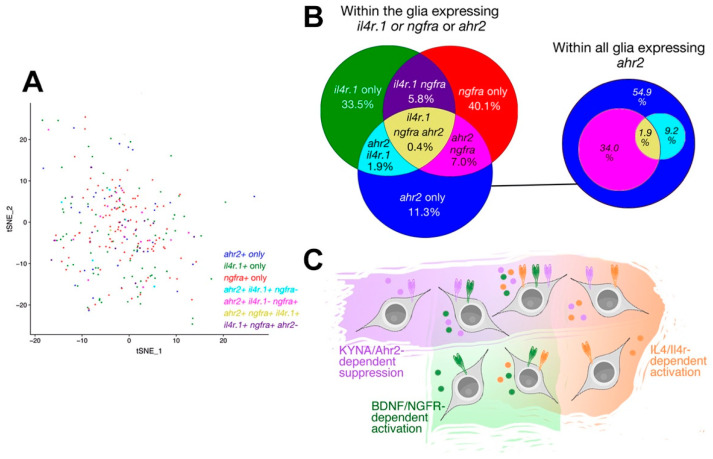
Single cell sequencing-based co-expression of *il4r*, *ngfra*, and *ahr2* in adult zebrafish brain. (**A**) tSNE plot for astroglial cells in adult zebrafish telencephalon showing the cells expressing *ahr2, il4r, ngfra* and all possible triple combinations. (**B**) Venn diagram for the abundance of progenitor cells expressing different combinations. (**C**) Schematic depiction of various subtypes of neural stem cells that are responsive to multiple signaling pathways. KYNA/Ahr2-dependent regulation marks a new functional subtype of neural stem cells in zebrafish brain.

By using bulk and single cell RNA sequencing, we previously identified two subtypes of zebrafish telencephalic neural stem cells (NSCs) that are responsive to Interlukin-4 (IL4) through its receptor Il4r, and to BDNF through its receptor Ngfra [1,2]. These findings indicated that zebrafish NSCs are heterogenous and employ different mechanisms for inducing their plasticity and neurogenesis responses [48]. To determine if Ahr2-expressing NSCs are overlapping with *il4r-* or *ngfra*-expressing NSCs, we prepared a tSNE single cell plot with existing single cell data on astroglial cells of the adult zebrafish telencephalon [1,3,4] and marked expression of three receptors: *ahr2*, *il4r* and *ngfra* (Figure 5A). We found glial cells that express only one of these receptors or different possible combinations of all three receptors (Figure 5A). We found that within all glia expressing any of the receptors, 33.5% express only *il4r*, 40.1% express only *ngfra*, and 11.3% express only *ahr2* (Figure 5B). The ratio of glia that expresses all three receptors simultaneously is 0.4%. Combinations of two receptors constitute 14.7% of the glia. Among all *ahr2*-expressing glia, 54.9% express neither *il4r* nor *ngfra* (Figure 5B). Furthermore, 34% of *ahr2*-positive glia also express *ngfra*, and 9.2% express *il4r*. These results indicate that *ahr2*-expressing glia constitute a new subtype of NSCs that is distinct from previously identified *il4r* or *ngfra*-positive glia, while a smaller portion of glia co-express either of these receptors with *ahr2* (Figure 5C).

Since KYNA/Ahr2 signaling is associated with the response of the zebrafish brain to amyloid toxicity and Alzheimer-like pathology, we investigated whether the genes involved in tryptophan metabolism were dysregulated in AD by performing TWAS within the brain autopsy three cohorts: (1) the Religious Orders Study and Memory and Aging Project (ROS/MAP); (2) the Mayo Clinic RNAseq cohort; and (3) the Columbia University Hispanic Brain Bank cohort [5] (Table 1). We selected the following genes from the KEGG pathway compilation for tryptophan metabolism (https://www.genome.jp/pathway/map00380+M00038, accessed on 12 July 2021) and Ahr signaling molecular interaction prediction (https://genemania.org/search/homo-sapiens/Ahr, accessed on 12 July 2021): *AADAT, AHRR, AIP, ARNTL2, EPHX1, HSP90AA1, HSP90AB1, KMO,* and *PTGES3.* The transethnic metanalysis revealed that KMO (kynurenine 3-monooxygenase), which converts L-Kynurenine to 3-Hydroxy-L-Kynurenine and reduces the availability of the substrate for kynurenic acid, is strongly associated with AD in humans with a positive false discovery rate (*p*_FDR) of 6.35 × 10^−5^ and consistent directionality (Table 1). Ahr interaction partners HSP90AB1 [49] and ARNTL2 [50] are also associated with AD. We also found that the enzyme AADAT (Kynurenine Aminotransferase II, KAT2), which we previously shown to be upregulated in AD patients’ brains and in an Alzheimer model of 3D cultures of human cortical networks [8], as well as in this paper, is also associated with AD in humans.

## 4. Discussion

**Figure 6:**
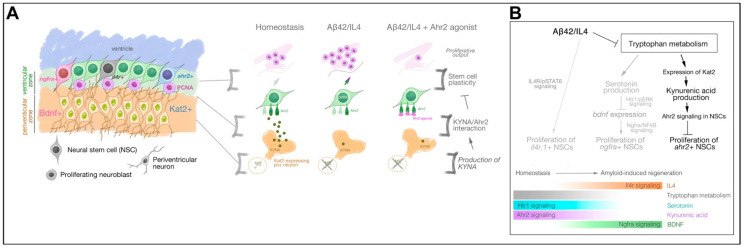
KYNA/Ahr2 signaling defines the third distinct Alzheimer’s disease-responsive subset of neural stem cell population in adult zebrafish brain (**A**) Schematic view of the regulation of neural stem cell plasticity and neurogenesis in adult zebrafish brain by KYNA/Ahr2 signaling. (**B**) Consolidated view of the effects of Aβ42 on neural stem cell plasticity. Aβ42 reduces the expression levels of KAT2, which generates KYNA, an inhibitory regulator of neural stem cell proliferation through its receptor Ahr2. For successful regeneration to take place, tryptophan metabolism is suppressed and IL4/BDNF-mediated signaling mechanisms are enhanced.

Zebrafish have the remarkable ability to regenerate their brains through enhanced neurogenesis upon injuries and neurodegeneration. Here, we report a specific regulation of tryptophan metabolism by amyloid toxicity in zebrafish brain, where the production of kynurenic acid (KYNA) is reduced by amyloid toxicity to allow for proliferation of a subset of NSCs that express aryl hydrocarbon 2 (Ahr2/AHR2, also referred as Ahr/AHR in humans) (Figure 6A). We found that KAT2 enzyme is expressed in the adult zebrafish brain in subventricular regions during homeostatic conditions and regulates the proliferation of neural stem cells. Amyloid toxicity suppresses production of KYNA, and thereby enhances cell proliferation. Ahr2 signaling agonists suppress cell proliferation in the absence of KYNA. We therefore suggest that Ahr2 signaling is a negative regulator of neural stem cell plasticity and neurogenesis in adult zebrafish brain upon amyloid toxicity. Combined with our previous findings, Ahr2 signaling constitutes the third method of regulation of neural stem cell plasticity in AD models in zebrafish brains, and signifies the importance of tryptophan metabolism for pathology-induced neurogenesis (Figure 6B). Our working model proposes that in the homeostatic state, tryptophan catabolism keeps KYNA/Ahr2 and serotonin/Htr1 signaling active, while amyloid-induced neuropathology suppresses the tryptophan metabolism through Interleukin-4, which leads to potentiation of BDNF/Ngfra signaling in NSCs and induces direct regulation of NSC plasticity through IL4/Il4r signal transduction [1,2,3,4,9,10,11,12,51] (Figure 6B).

In 3D human cortical neuronal cultures of the amyloid toxicity model, IL4 treatment significantly reduced KAT2 expression and production of KYNA [8]. Therefore, our results in this manuscript recapitulate the regulatory mechanism in human cells. Furthermore, when we injected KYNA into adult zebrafish brains, we found that it significantly reduced the stem cell proliferation (Figure 1). In an *APP/PS1* mouse model of AD and postmortem human brains with AD, *KAT2* expression was upregulated in stem cells and neurons after amyloid deposition [25]. We indeed found that in mouse models of AD and in post-mortem brains of AD patients, KYNA levels were upregulated, suggesting that elevated KYNA levels might be detrimental to NSC proliferation and neurogenesis in vivo. These results are consistent with the serum levels of KYNA in AD patients, where KYNA levels correlated with TAU hyperphosphorylation and inflammation biomarkers [52]. These findings further supporting our hypothesis that KYNA has an evolutionarily conserved activity of suppressing neural stem cell proliferation in vertebrates.

The expression levels of *KAT2* reduce in glioma [53,54,55], and this serves as an advantage for the tumor to escape proliferative regulation as KYNA inhibits the proliferation and migration of human glioblastoma [15]. Our results suggest that (1) the regulation of kynurenic acid levels in vertebrate brains with AD could be an evolutionarily conserved mechanism; and (2) kynurenic acid negatively affects neural stem cell plasticity and neurogenic ability in vertebrate brains during AD. Interestingly, KYNA has also been shown to be a neuroprotective agent. Direct application of KYNA to primary cortical neurons positively affects neuronal outgrowth, complexity, and synapse formation in vitro [55,56]. When combined, the results suggest an interesting hypothesis that KYNA may have cell-specific roles: suppressing neural stem cell plasticity and neurogenic ability and promoting neuronal synaptic connectivity. This role of KYNA suggests that this molecular program could have cell-type-specific roles during the course of AD.

KYNA binds to four types of receptors: G-protein-coupled receptor 35 (GPR35), Aryl-hydrocarbon receptor (Ahr or AHR), Glutamatergic receptors (AMPA, NMDA and KAR), and alpha7-nAcetylcholine receptor (a7nAChR) [17,18,49]. KYNA is a direct agonist for GPR35 and reduces the intracellular Ca^+^, cAMP, AKT/ERK/p38 phosphorylation and elevated beta-catenin levels [57,58,59,60]. KYNA activates the dimerization of pro-transcription factor Ahr and its nuclear translocator (ARNT) to generate an active transcriptional complex, which regulates NFkB signaling pathways [61,62], and is an antagonist for neurotransmitter signaling as it can inhibit NMDA and AMPA receptors [63,64,65]. Therefore, the effect of KYNA on respective tissues is highly versatile depending on the receptor type expressed in the target tissue. Our single cell study indicated that Ahr2 is the only receptor expressed in neural stem cells in adult zebrafish brain. This is consistent with previous findings in a traumatic injury model of zebrafish brain [66] both for signaling route in neural stem cells and for the effects of Ahr signaling on proliferation and neurogenesis.

Our TWAS studies showed a clear association of KYNA/Ahr signaling in humans with AD (Table 1); however, the nature of this association and how KYNA/Ahr signaling axis would affect AD pathology is not fully clear. In the clinical setting, KYNA is being used as a positive agent towards the treatment of AD, because KYNA can hamper AMPA signaling and alleviate excitotoxicity [18,25,67,68,69]. With our findings in zebrafish, an alternative understanding of the longitudinal involvement of KYNA signaling in AD pathology in humans can be that the modulation of KYNA could be re-structured in AD brains to allow stem-cell-based regenerative neurogenesis. It is probable that at early disease stages where KYNA is low, NSCs can still perform, while at later stages of disease pathology, KYNA production is elevated (consistent with the TWAS data in Table 1 as *KMO* is underexpressed, which increases the availability of L-Kynurenine to be converted to KYNA) and NSC proliferation is reduced. Additionally, since the directionality of *AADAT* in human TWAS analysis tends towards underexpression, this might also mean that the AD brains in humans try to downregulate KYNA/Ahr signaling, yet the net outcome does not yield enhanced neurogenesis due to other confounding factors.

In our study, we focused on astroglial cells that act as neural stem cells in zebrafish. However, AHR signaling also regulates the immune system and immune cell physiology [70]. Given that immune–glia crosstalk is a significant factor that determines the neurogenic outcome in zebrafish [19,21,22,26,30,35,71], a potential continuation of our work would be to investigate the role of Ahr signaling on immune cells in the zebrafish brain during AD, and possible secondary effects of the altered immune response on neurogenesis.

Overall, we suggest that changes in the neurogenesis rate in AD might correlate with the antagonistic activities of Interleukin-4 and tryptophan metabolism partially through the KYNA/Ahr signaling axis. This hypothesis requires further experimental evidence but could help explain the reduced neurogenesis in human brains as AD pathology progresses. Further experimental models and clinical data are likely to provide a more in-depth understanding of the relationship between neurogenesis and AD, as well as the underlying molecular mechanisms.

## Figures and Tables

**Table 1:** Transcriptome-wide association study (TWAS) for AHR-signaling-related genes in human LOAD cohorts.

Gene	Meta Expression	*p*-Value	DGE Direction	*p*_FDR
*KMO*	−0.2009332	3.18 × 10^−6^	−−−	6.35 × 10^−5^
*AHRR*	−0.1683677	0.000283	−−−	0.0018
*HSP90AB1*	−0.0585899	0.004116	+−−	0.01456
*ARNTL2*	−0.05694	0.02121	−−−	0.0531
*AADAT*	−0.0449039	0.0249	−−−	0.06025
*EPHX1*	0.0643375	0.08271	+++	0.1542
*AIP*	0.0208076	0.2023	+−+	0.3095
*HSP90AA1*	0.0092185	0.7284	+++	0.8048
*PTGES3*	−0.0016771	0.9305	+−+	0.9536

“DGE direction” shows the differential gene expression direction for genes that are overexpressed (“+”) or underexpressed (“−”) in cases vs. controls. “*p*_FDR” shows the genome-wide FDR adjusted *p*-values (in bold are those genes that are genome-wide significant).

## Data Availability

Zebrafish transcriptomics data is available with GEO accession numbers: GSE74326, GSE118577, and GSE124162. Alzheimer’s disease cohort data is retrieved from AD Knowledge Portal (https://adknowledgeportal.org, accessed on 26 August 2021).

## Supplementary Materials

The following are available online at https://www.mdpi.com/article/10.3390/cells10102748/s1, Table S1: Quantification data for figures. All data points, values and statistical analyses for corresponding figure panels are provided in this table. Table S2: Information about the agonist, antagonist, antibodies, and primers are provided in this table.
